# Which factors influence orthodontists in their decision to extract? A questionnaire survey

**DOI:** 10.4317/jced.55709

**Published:** 2019-05-01

**Authors:** Astrid Evrard, Michele Tepedino, Paolo M. Cattaneo, Marie A. Cornelis

**Affiliations:** 1 Private practice, Couillet, Belgium; 2 Department of Biotechnological and Applied Clinical Sciences, University of L’Aquila, Italy; 3 Associate Professor, Section of Orthodontics, Department of Dentistry and Oral health, Aarhus University, Faculty of HEALTH, Aarhus, Denmark

## Abstract

**Background:**

To evaluate the relative influence of different criteria in the choice between extraction and nonextraction treatment in current orthodontics, and to assess how the percentage of extractions has evolved over time.

**Material and Methods:**

Pre-treatment records (panoramic radiograph, lateral cephalogram, study casts and photographs) of fourteen cases in permanent dentition (adult or adolescent) with class I molar relationship and moderate anterior crowding were evaluated by 28 orthodontists. For each case, each orthodontist filled out a questionnaire reporting his treatment plan proposal (extraction or nonextraction) and the importance of specific parameters in his decision-making process, using categorical scales. Orthodontists practicing for more than 15 years were also asked to compare this decision with the one they would have taken at the beginning of their professional career.

**Results:**

The two most important factors in the decision-making were the soft tissue profile and the amount of crowding. The least important factor was the presence of third molars. In cases of nonextraction treatment, the lack of space was managed mostly by dental expansion and stripping. Twenty percent of the case evaluations revealed extraction(s) decisions. Among the orthodontists practicing for more than 15 years, the current extraction rate reached 24%, whereas the same orthodontists reported they would have extracted in 39% of the cases in the past.

**Conclusions:**

The present study suggests that soft tissue profile has a higher impact than traditional criteria such as cephalometric measurements in the extraction decision. This is associated with a decreased extractions rate compared to the past.

** Key words:**Orthodontics, extractions, survey, treatment planning.

## Introduction

The discussion about extractions in orthodontics started in the early 1900s, when Angle argued in favor of nonextraction treatments: he believed that orthodontic forces would be associated with growth and achieving of alignment of the whole dentition (1). Tweed, Angle’s disciple, initially disagreed with Case, who believed that nonextraction treatment often led to a lack of stability (2). Tweed was eventually discouraged by the resulting protrusive faces and treatment relapse. He decided to retreat some patients with extractions and concluded that carefully and consistent planned extractions allowed him to improve patient’s appearance as well as treatment stability (3). For these reasons, in the mid-1940’s, extractions in the permanent dentition became the most common treatment approach to correct malocclusions. The idea of “the diagnostic facial triangle”, based on the statement that incisors need to be uprighted over the basal bone to reach harmony and facial balance (4), became in vogue. Currently this polemic is ongoing, but for many reasons extractions are used less than in the past, when they were dictated by cephalometric measurements. The quality of orthodontic treatment improved with the arrival of bonding techniques (5), exploitation of growth potential started being used (6-10), and extractions were shown not to guaranty stability (11). The impact of extractions on facial esthetics began to be questioned: some investigators claimed that extractions produced a flat soft-tissue profile “dishing in the lips” relative to the chin and nose (12,13), while others indicated that it was simplistic to blame extractions solely for unaesthetic results (14-18).

There is a conspicuous lack of evidence to support either position in the extraction debate (19). For borderline patients, clinicians use their diagnostic tools such as cephalometric analysis, models and photographs; but the final decision remains subjective and clinical experience is used to decide the treatment plan for the most appropriate outcome. Due to the scarcity of scientific evidence, understanding the specific diagnostic parameters influencing orthodontists in their treatment planning is important. The purpose of the present study was to evaluate which criteria clinicians use to choose to extract or not to extract. In addition, we examined how the percentage of extractions has evolved over the past 15 years.

## Material and Methods

Thirty certified orthodontists were randomly selected among the alumni of the Université catholique de Louvain, Belgium, for a questionnaire survey. Twenty-eight agreed to participate. Half of them were clinicians having more than 15 years of experience (23.4±4.9 years) and the others had less than 15 years of experience (7.5±3.2 years).

The orthodontists were asked to establish the treatment plan, with or without extractions, of fourteen untreated cases with class I canine and molar relationships and moderate anterior crowding (2.6±1.3mm [range 0.5-4.5]). All study subjects were skeletal class I, in permanent dentition with all teeth fully erupted, no dental anomalies, congenitally missing or extracted teeth. The 14 cases were divided into an adolescent (4 males and 4 females; 13.1±2.1 years of age [range 10-16]) and an adult (3 males and 3 females; 29.5±7.1 years of age [range 22-40]) cohort. The records given to the orthodontists for their evaluation included pre-treatment study casts, panoramic radiograph (OPT), lateral cephalogram and facial (frontal, profile and three-quarter) as well as intraoral photographs. In order to speed up the analysis, the amount of crowding had been previously quantified for each case. The orthodontists were also asked to evaluate the importance of 9 parameters in their decision-making process for each case (crowding, overbite, midline deviation, arch and teeth shape, curve of Spee, presence of third molars, vertical skeletal pattern, axes of upper and lower incisors and facial profile) on 5-point scales (0 : no importance for treatment planning - 4 : high importance for treatment planning), in order to reflect the importance of each factor in their decision to extract or not. In the case of an extraction treatment plan, the orthodontists were asked which teeth they would have extracted. When a nonextraction treatment plan was chosen, they were asked how they would manage the lack of space (e.g. dental expansion, skeletal expansion, stripping or distalization). The experienced clinicians (>15 years of practice) were also asked for each case if they would have made the same decision at the beginning of their career. Finally, the questionnaire asked the orthodontists about the types of brackets and retention they used at the beginning of their career and at the time of the survey.

Categorical scales were used to determine the importance of the different parameters. Frequencies and standard deviations were calculated. Among the experienced orthodontists group, a McNemar test was used to compare the past and present extractions decisions. Statistical significance was set at *p*≤ 0.05.

## Results

The 392 case evaluations (14 cases examined by 28 orthodontists) revealed 313 nonextraction treatments (80.0%) and 79 extraction treatments (20.0%). When the 14 experienced orthodontists (>15 years of experience) were asked if they would have made the same decision at the beginning of their career, the percentage of extraction treatments increased to 39% at the start of their career, which was significantly higher than the percentage of extractions they would have recommended today (24%) (*p*<0.001).

**Figure 1:**
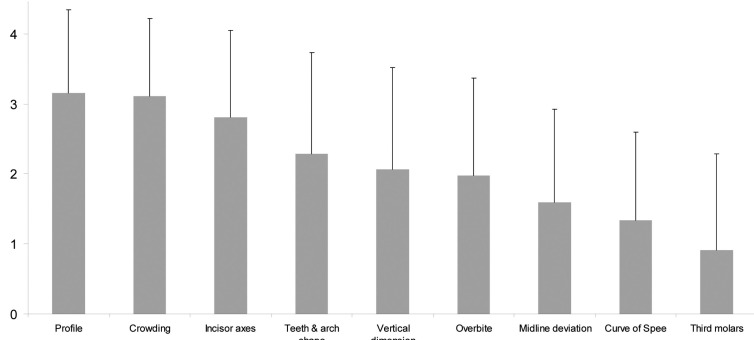
Importance of evaluated parameters for treatment planning (0: no importance for treatment planning – 4 : very important for treatment planning).

**Figure 2:**
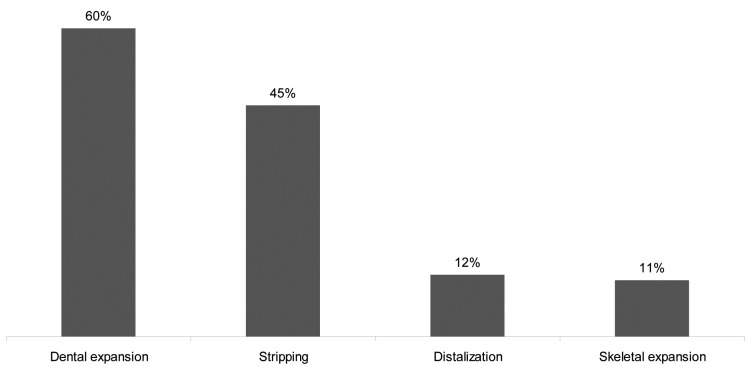
Nonextraction treatments: distribution of frequencies of methods used for space management (orthodontists could choose more than one).

**Figure 3:**
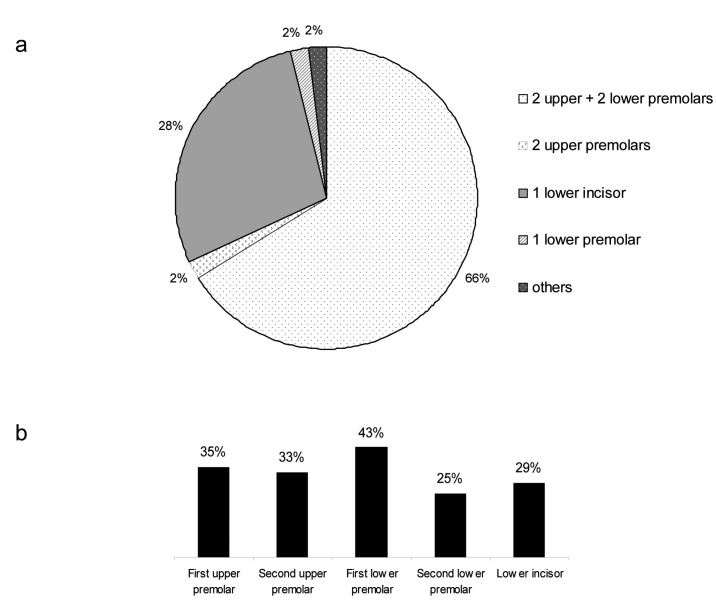
Extraction treatments: Patterns of combined extractions in the upper and lower jaws (a); Distribution of frequencies of individually extracted teeth (b).

Figure 1 shows the importance of soft tissue profile, crowding, incisor axes, tooth and arch shape, vertical dimension, overbite, midline deviation, curve of Spee, and presence of third molars in the extraction/nonextraction decision process. The two most important factors were the soft tissue profile and the amount of crowding. The presence of third molars was the least important factor. In nonextraction treatments, the lack of space was managed mostly by dental expansion and stripping (Fig. 2). In case of extraction treatments, the most common pattern of extractions was four premolars extractions. The distribution of these patterns is shown in Figure 3a, and the distribution of individually extracted teeth is shown in Figure 3b.

**Figure 4:**
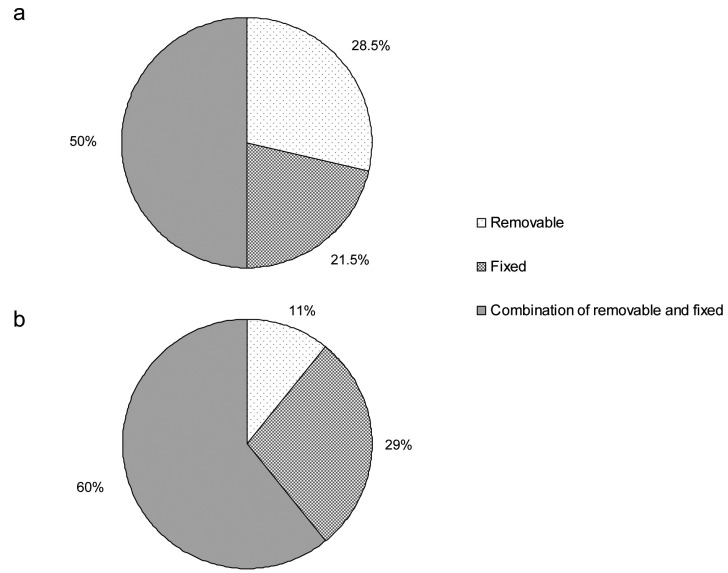
Types of retention used by the orthodontists at the beginning of their career (a) and at the time of the survey (b).

Self-ligating brackets were reported to be used by half of the clinicians at the time of the survey (although only 2 among them used them exclusively), while no orthodontists used self-ligating brackets at the beginning of their practice. The types of retention (removable, fixed or a combination of removable and fixed) used by the orthodontists are shown in Figure 4. Orthodontists tend to use less removable retention appliances currently as compared to their early career. However, a lower bonded and upper removable retainer was still the most reported combination within the sample at the time of the survey.

## Discussion

Most orthodontists agree that the decision about whether to extract is too important to be left to intuition alone. Even senior clinicians are not immune from making mistakes despite their wealth of experience. The consequences of two types of wrong decisions are different: while failure to extract where required may almost always be corrected later on, a wrong decision to extract leaves no margin for later correction (12).

In the present study, 79 out of 392 decisions lead to extraction treatments, and our data find that more extractions would have been recommended by these same clinicians 15 years ago. The reasons given by the surveyed orthodontists for this reduced extraction rate was the development of new techniques, such as self-ligating brackets and skeletal expansion. Additionally, orthodontists are trained dentists who are encouraged to preserve teeth (20). The rate of extraction treatments in the present study was close to that reported by Jackson et al. when retrospectively evaluating the treatment modality in a University Clinic (21). They observed an overall extraction rate of 25%, and a four premolars extraction rate of 13%, although their orthodontic population comprised patients with any kind of malocclusions and not only Class I (21).

The orthodontists were asked to score the parameters influencing their decision-making process towards extraction or nonextraction. Crowding was confirmed to be an important parameter in their decision, but unexpectedly, it did not reach the highest score, and this is in accordance with other studies (21). The major factor dictating extractions was the soft tissue profile (Fig. 1), thus highlighting orthodontists’ concern about facial and smile esthetic appearance (22). Surprisingly, the prevalence of extractions of first and second upper premolars was very similar (Fig. 3b), which seems to contradict the importance given to profile, since several surveyed orthodontists commented that they were concerned about the impact of first versus second premolars extractions on lip response. There are conflicting reports on the response of soft tissues following changes at the hard tissue level. Some investigators reported a direct relationship between them (23-26), whereas others came to the conclusion that the behavior of soft tissue profile is independent of hard tissues (14,17-18), potentially because of the flexible and mobile nature of the lip texture (27). This issue draws the attention of orthodontists, as the effect of orthodontic treatment on the face continues to be debated: there is general agreement that orthodontic treatment can influence the soft-tissue profile, but there is still disagreement on the amount of soft tissue response to the changes in tooth position and alveolar process position (16,24,26). The third most reported reason for extractions was the inclination of the incisors’ axes. Although the literature is lacking sound evidence that proclined teeth have an increased risk of gingival recession (28), this is still considered an issue in some cases for the soft tissue profile and facial aesthetics (22).

Although Tweed claimed that low angle patients might not need as much mandibular incisor uprighting as patients with a higher angle (4), vertical skeletal pattern did not appear to play a major role in the extraction decision in the present study. Crowding in nonextraction cases can be solved by molar distalization, but this increases the vertical dimension of the face. Merrifield and Cross reported that for every millimeter of vertical extrusion in the molar area, a 1.3 mm increase in anterior facial height occurs (29). The downward and backward mandibular rotation may thus be followed by an increased gingival display on smiling and a poor esthetic result, except in patients with deep bites and reduced vertical dimension (30). There exists a paradox between the importance of the impact of profile and esthetics and the apparent lack of significance of the vertical skeletal pattern in the extraction decision. On the other hand, this is in accordance with the results of a systematic review, that concluded that the extractions of four premolars do not have a significant effect on facial profile (31).

The presence of wisdom teeth was not considered by the orthodontists as a determinant factor for treatment planning (extraction/nonextraction) in the present study. Indeed, extraction of third molars often is a therapeutic decision for prevention of future pathology or impaction rather than crowding relapse, as crowding may relapse even if wisdom teeth are absent (32). Interestingly, most of the orthodontists in this study consider a treatment as nonextraction even if third molars need to be extracted after debonding.

The most frequently reported techniques used for gaining space in nonextraction cases were (as expected) dental expansion and stripping (Fig. 2). Distalization was used in only 12% of the cases, which is probably related to the fact that the cases presented a class I molar relationship. Interestingly, skeletal expansion, meaning symphyseal and palatal distraction, was mentioned in 11% of the cases, which seems to be relatively high regarding to the invasiveness of the surgical technique.

The conventional brackets remained the most prevalent in the present study. Self-ligating, “low-friction” brackets are claimed to allow greater amounts of dental expansion, and thus to decrease the need for extractions (33), which might also be a reason for a decreased extraction rate compared to the past.

The most noticeable finding about retention devices was the decreased use of removable appliances, although a combination of bonded retention in the lower jaw and removable retainer in the upper jaw was still the most used system at the time of the survey. One possibility is that in the past removable retention appliances had no efficient alternative, whereas currently bonded retainers are easier and more predictable than ever before, especially when patients’ cooperation is poor. By contrast, bonded retainers increase the risk of emergencies in the practice. In these conditions, a combination of a lower bonded retainer and an upper removable one seems to be a good compromise, as the stability of the upper teeth is ensured by contact with the lower ones, which need to be stabilized in the long term since they sustain a constant pressure that if unbalanced reduces the intercanine distance (34-35).

In the present study, we aimed to keep the conditions as similar as possible to clinical practice: all records were provided (OPT, cephalogram, photographs and models). Each clinician prepared his treatment plan independently, deciding whether extraction or nonextraction was preferable for each individual case. This approach, appreciating the orthodontists’ actual decision-making procedure under clinical conditions, might rationally be considered superior to an alternative evaluation where they would summarize their theoretical positions in general (36). For expediency, the orthodontists were given multiple-choice questionnaires, but they were given the option to add comments. They had one hour to fill out the questionnaires; this might have been too little time alotted to analyze the records like they would have done for their own patients, and could be considered a weakness of the study. On the other hand, asking them more time could have limited the number of orthodontists willing to participate. In general, the surveyed clinicians overall used their clinical experience to treatment plan, as they do in their practice.

## Conclusions

-The two most important parameters influencing the extraction/nonextraction decision are the soft tissue profile and the amount of crowding.

-Nonextraction treatment’s space discrepancy is mostly managed by dental expansion and stripping.

-The rate of extractions shows a decreased tendency as compared to the past, most likely related to the technical improvements of contemporary orthodontics.
